# Management of Ovarian Dermoid Cysts by Laparoscopy Compared With Laparotomy

**DOI:** 10.1155/DTE.3.19

**Published:** 1996

**Authors:** Pang Liyi, Hiroshi Sasaki, Liu Chang Qing, Minoru Akiyama, Akihiko Watanabe, Shigeki Niimi, Tadao Tanaka

**Affiliations:** Department of Obstetrics and Gynecology The Jikei University School of Medicine 3-25-8 Nishi-shinbashi Minato-ku Tokyo 105 Japan

## Abstract

Thirty patients with ovarian dermoid cysts removed by laparoscopic surgery were compared with 42 patients with ovarian dermoid cysts removed by laparotomy, with respect to the selection criteria, surgical procedures, operating time, intraoperative and postoperative complications, blood loss, and hospital stay. Although the operating time for unilateral cystectomy, unilateral salpingo-oophorectomy, and bilateral cystectomy performed by laparoscopic surgery was longer (120.3 ± 43.7 min, mean ± SD) than those for the same procedures performed by laparotomy (73.9 ± 21.6 min, *p* < 0.01), we observed a learning curve with a remarkable declining tendency (linear regression model, *p* < 0.01). At the end of this study, the times taken for laparoscopic procedures were almost the same as those for laparotomy. Less blood loss (18.2 ± 1.7 ml versus 105.9 ± 84.3 ml, *p* < 0.01) and shorter hospital stay (5.9 ± 1.9 days versus 12.0 ± 2.9 days, *p* < 0.01) were also found to be advantages of laparoscopic surgery. This article discusses the technical procedures of laparoscopic surgery. The efficiency and safety of operative laparoscopy as an alternative access route for the management of ovarian dermoid cysts were recognized. We stress that strict criteria for selection of patients should always be followed and the necessity of retraining schedules for gynecologists and nursing staff in the speciality of laparoscopic surgery.


*Diagnostic and Therapeutic Endoscopy*, Vol. 3, pp. 19–27
Reprints available directly from the publisher
Photocopying permitted by license only

© 1996 OPA (Overseas Publishers Association)
Amsterdam B.V. Published in The Netherlands
by Harwood Academic Publishers GmbH
Printed in Singapore

### Management of Ovarian Dermoid Cysts by Laparoscopy Compared with Laparotomy

PANG LIYI, HIROSHI SASAKI*, LIU CHANG QING, MINORU AKIYAMA, AKIHIKO WATANABE, SHIGEKI NIIMI and TADAO TANAKA

*Department of Obstetrics and Gynecology, The Jikei University School of Medicine, 3-25-8 Nishi-shinbashi, Minato-ku, Tokyo 105, Japan*

*(Recieved 28 February 1996; In final form 1 March 1996)*

**Thirty patients with ovarian dermoid cysts removed by laparoscopic surgery were compared with 42 patients with ovarian dermoid cysts removed by laparotomy, with respect to the selection criteria, surgical procedures, operating time, intraoperative and postoperative complications, blood loss, and hospital stay. Although the operating time for unilateral cystectomy, unilateral salpingo-oophorectomy, and bilateral cystectomy performed by laparoscopic surgery was longer (120.3 ± 43.7 min, mean ± SD) than those for the same procedures performed by laparotomy (73.9 ± 21.6 min, $p < 0.01$), we observed a learning curve with a remarkable declining tendency (linear regression model, $p < 0.01$). At the end of this study, the times taken for laparoscopic procedures were almost the same as those for laparotomy. Less blood loss (18.2 ± 1.7 ml versus 105.9 ± 84.3 ml, $p < 0.01$) and shorter hospital stay (5.9 ± 1.9 days versus 12.0 ± 2.9 days, $p < 0.01$) were also found to be advantages of laparoscopic surgery. This article discusses the technical procedures of laparoscopic surgery. The efficiency and safety of operative laparoscopy as an alternative access route for the management of ovarian dermoid cysts were recognized. We stress that strict criteria for selection of patients should always be followed and the necessity of retraining schedules for gynecologists and nursing staff in the speciality of laparoscopic surgery.**

*Keywords:* Dermoid cyst, extraperitoneal resection, intraperitoneal resection, laparoscopic surgery, ovarian neoplasm, retrieval bag

### INTRODUCTION

Operative laparoscopy has become one of the most common applications in the management of ovarian cysts. According to the survey of the American Association of Gynecologic Laparoscopists (AAGL) in 1990, a total of 13,739 laparoscopies were performed for this indication in United States, and 70% of patients with persistent ovarian masses were managed by laparoscopic surgery alone [1]. Laparoscopic surgery has been reported to have potential advantages over laparotomy, such as small wound, short

*Corresponding author. Tel.: +81-3-3433-1111 (ext 3520). Fax: +813-3-3433-1111 (ext 3527).

operating time, less complications, rare adhesion formation, lower overall cost of care, and rapid convalescence [2,3]. In the investigation of adnexal masses, ovarian dermoid cysts are often encountered, and account for about 10 to 44% of ovarian neoplasms [4,5]. The key points in laparoscopic removal of dermoid cysts are proper preoperative selection of patients and avoidance of intraoperative spillage, which makes this procedure controversial in cases of malignancy [6]. Since almost all laparoscopic research related to ovarian cysts has put the emphasis on the laparoscopic procedure only and paid little attention to its performance in comparison with laparotomy with respect to its advantages and pitfalls in clinical practice, proper evaluation in this regard is urgently required [7]. This retrospective study reviews our experiences in the laparoscopic removal of dermoid cysts over 19 consecutive months, in comparison with laparotomies over the same period. The selection criteria, advantages, and procedures of laparoscopic surgery are discussed.

### MATERIALS AND METHODS

### Patients

From October 1993 through April 1995, 30 patients with a preoperative diagnosis of ovarian benign dermoid cyst underwent laparoscopic surgery at the Jikei University Hospital. The selection criteria for this procedure included 1) no personal or family history of gynecologic, breast, and colorectal cancer, 2) typical features of dermoid cyst with no sign of malignancy on pelvic examination, transvaginal ultrasound, and computed tomography or magnetic resonance imaging, 3) levels of tumor markers such as CA125 and CA19-9 within normal limits, and 4) preoperative counseling including information about the risk of complications as well as the possibility of laparotomy in cases of suspected malignancy. Consent was obtained from each patient or her family after making sure they had a full understanding of this procedure.

Another 42 patients underwent removal of ovarian dermoid cyst by laparotomy in the same period. Some of them were found not to be appropriate candidates for laparoscopic procedure and some were seen by doctors in our department who did not perform laparoscopic surgery. All of them were extensively investigated, and benign dermoid cyst was diagnosed preoperatively. The histopathologic diagnosis was benign ovarian dermoid cyst in all patients in the two groups.

All of the patients were reviewed with respect to the indications, methods, operating time, intraoperative and postoperative complications, blood loss, and hospital stay.

### Laparoscopic Surgery

The procedure was performed under general anesthesia with endotracheal incubation. The patient was placed in the lithotomy position, and the bladder was catheterized. Open laparoscopy was commenced by making a 2- to 3-cm transverse infraumbilical incision and dissecting into the peritoneal cavity. A 12-mm trocar (Ethicon Endo-surgery, a Johnson & Johnson Company, Cincinnati, OH) was inserted according to Hasson's method [8]. After establishment of a carbon dioxide pneumoperitoneum, the patient was repositioned in the deep Trendelenburg position. The laparoscope was then introduced through this trocar. With the aid of moving a Hegar's dilator placed in the uterine cavity, the entire peritoneal cavity was carefully inspected to determine the feasibility and procedure of laparoscopic surgery. Another two trocars (12 and 5 mm) were inserted in the lower abdomen lateral to the inferior epigastric vessels on each side. Occasionally a fourth trocar was inserted in the suprapubic area. The suction-irrigator probe (Probe Plus II, Ethicon Endo-surgery) was inserted to aspirate ascites or peritoneal washings for cytologic examination, using a 30-ml syringe. Division of adhesions was performed with unipolar or bipolar cautery, hooked scissors, or a combination of these instruments. The decision to perform extraperitoneal cystectomy, intraperitoneal cystectomy, or salpingo-oophorectomy by laparoscopic surgery was made according to the patient's age, desire for children, and the nature of the cyst.

### *Extraperitoneal Cystectomy*

This procedure was limited to patients who were unmarried or nulligravida if sufficient ovarian tissue could be preserved. An isolating bag (usually used for isolating intestine, 48 × 48 cm, made by 3M Health Care) was cut at an angle according to the tumor size, and a small amount of saline was put into it. The bottom of the bag was held with a pair of atraumatic forceps and inserted through one 12-mm trocar sleeve. The bag was pulled completely inside the abdomen and opened by separating the two top edges with the help of another pair of forceps. The tumor was then placed in the bag. A 3- to 5-mm incision in the cyst wall was made with a unipolar electrode inside the suction-irrigator probe, which was inserted into the cyst through the incision. Then the cyst contents were aspirated thoroughly while the cyst was irrigated with warm saline. The top edges of the bag were held together by claw forceps and brought up to the abdominal incision while the forceps was rotated in one direction. In most cases it was easy to pull the cyst with reduced size out of the abdomen completely, and under direct vision cystectomy was performed as with laparotomy [9,10]. The preserving ovary was returned to the peritoneal cavity. In some cases it was necessary to remove the remaining contents of the cyst through the abdominal incision until the cyst was small enough to be taken out. Enlargement of the abdominal incision due to a large solid tumor content was rarely needed. This procedure was performed in 19 patients.

### *Intraperitoneal Cystectomy*

This method was used mainly for small cysts that could not be extracted because of adhesion of the mass to the uterus, rectum, or abdominal wall. Cystectomy was performed by a combination of sharp and blunt dissection using hooked scissors and atraumatic forceps. The resected cyst was held in the bag and pulled to the abdominal incision. The contents were aspirated, and the solid part was removed with forceps and pulled out through the incision. Hemostasis was achieved by electrocautery. The remaining ovary was sutured under laparoscopy. We performed this kind of procedure in 4 patients including 1 bilateral and 3 unilateral cystectomies.

### *Salpingo-oophorectomy*

If it was thought that ovarian tissue could not be preserved or the patient was parous, then unilateral salpingo-oophorectomy was carried out. First, the ureter was identified along its free course. The infundibulopelvic ligament, ovarian ligament, and proximal fallopian tube were totally electrodesiccated and then cut with hooked scissors. The stump of the infundibulopelvic ligament was ligated again with Endoloop (Ethicon Endo-surgery) or in some cases, Endopath (Ethicon Endo-surgery). The adnexal tissue was placed into the bag, and subsequent procedures were the same as described previously.

One surgeon (H.S.) performed all laparoscopic surgery, but the assistants often differed. It was not possible to organize a special group of gynecologists and nursing staff to perform laparoscopic surgery. The surgeons performing laparotomy differed depending on who was responsible for the patient. The techniques used for laparotomy were similar in all cases.

### RESULTS

We performed operative laparoscopy in 30 patients and laparotomy in 42 patients with ovarian dermoid cysts in the same period. Table I shows the backgrounds of the two patient groups. The most common age group was 31 to 40 years in the laparoscopy group and 21 to 30 years in the laparotomy group, accounting for 43.3 and 57.1% of total cases, respectively. The age distribution of the laparoscopy and laparotomy groups showed no significant difference . Ovarian dermoid cysts occurred more frequently in the 20 to 40-year-old group, which is the reproductive period in women. No patients older than 60 or younger than 20 years were operated on by laparoscopic surgery; these patients were treated by laparotomy. Most (70 and 69%, respectively) of the ovarian masses were found during routine screening in both

TABLE I Background of Patients in Both Groups

| Subject | Laparoscopy ($n$=30) | Laparotomy ($n$=42) | $\chi^2$ test |
|---|---|---|---|
| Age | | | |
| ≤ 20 | 0 | 4 ( 9.5%) | |
| 21 – 30 | 9 (30.0%) | 24 (57.1%) | |
| 31 – 40 | 13 (43.4%) | 7 (16.7%) | |
| 41 – 50 | 5 (16.7%) | 4 ( 9.5%) | |
| 51 – 60 | 3 (10.0%) | 1 ( 2.4%) | |
| > 60 | 0 | 2 ( 4.8%) | $p > 0.05$ |
| Chief complaint | | | |
| Low abdominal pain | 7 (23.3%) | 7 (16.7%) | |
| Abdominal distention | 2 ( 6.7%) | 0 | |
| Dysmenorrhea | 0 | 6 (14.3%) | |
| Mass found at screening | 21 (70.0%) | 29 (69.0%) | $p > 0.05$ |
| Tumor markers | | | |
| CA125 normal range <35 U/ml | | | |
| Negative | 28 (93.4%) | 32 (76.2%) | |
| Positive | 2 ( 6.6%) | 10 (23.8%) | $p > 0.05$ |
| CA19-9 normal range <37 U/ml | | | |
| Negative | 28 (93.4%) | 36 (85.7%) | |
| Positive | 2 ( 6.6%) | 10(23.8%) | $p > 0.05$ |
| Tumor size | | | |
| < 5 cm | 8 (23.5%) | 4 ( 8.7%) | |
| 5 – 10 cm | 24 (70.6%) | 34 (73.9%) | |
| > 10 cm | 2 ( 5.9%) | 8 (17.4%) | $p > 0.05$ |
| Laterality | | | |
| Bilateral | 4 (13.3%) | 4 ( 8.6%) | |
| Left side | 11 (36.7%) | 19 (45.7%) | |
| Right side | 15 (50.0%) | 19 (45.7%) | $p > 0.05$ |

the laparoscopy and laparotomy groups, and the distribution of chief complaints also showed no significant difference between the groups. The rates of positivity for CA125 and CA19-9 in patients undergoing laparotomy (23.8 and 14.3%) were higher than the rates for those undergoing laparoscopy (6.6 and 6.6%), but the differences between the two groups were not significant. Most tumors measured 5 to 10 cm in both laparoscopy and laparotomy groups (70.6 and 73.9%). Tumors larger than 10 cm also could be resected by laparoscopic surgery without additional difficulties (5.9%). There was no significant difference in tumor size between the two groups. There was also no significant difference in laterality between the two groups. Thus, it was considered that the utility of the surgical techniques could be compared between the two groups. Because adhesion rarely occurred in cases of ovarian dermoid cyst, this factor was not analyzed.

The procedures performed via laparoscopy and laparotomy are shown in Figures 1 and 2. Unilateral cystectomy was the most common procedure (53.4%) in the laparoscopy group, whereas unilateral salpingo-oophorectomy was the most common procedure in the laparotomy group (47.7%), but there were also no significant differences between the two groups.

Table II shows the operating time, hospital stay, and blood loss in the two groups. The operating time was longer for laparoscopic surgery, but the difference between the two groups for each of the three procedures were not significant, which might be due to the small number of cases included in each subgroup. The total operating time for laparoscopic surgery was 120.3 ± 43.4 min (mean ± SD) compared with 73.9 ± 21.6 min for laparotomy, with a significant difference ($p < 0.01$, $\chi^2$ test). As shown in Figure 3, the operating time for laparoscopic surgery decreased remarkably with time; the correlation coefficient was significant

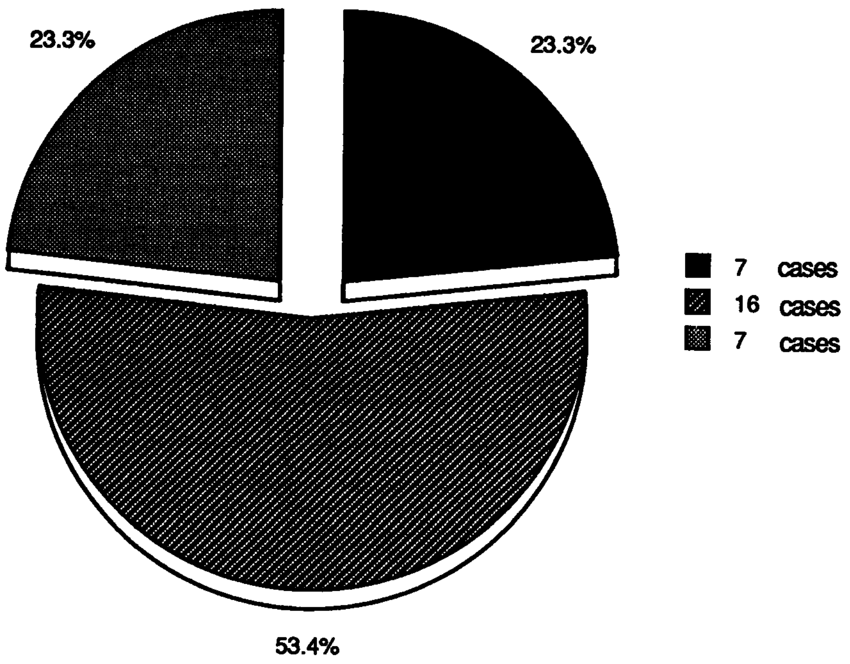

FIGURE 1 Patient distribution for three procedures performed by laparoscopic surgery: ■, unilateral cystectomy, 16 patients (53.4%); ■, bilateral cystectomy, 7 patients (23.3%); ■, unilateral salpingo-oophorectomy, 7 patients (23.3%).

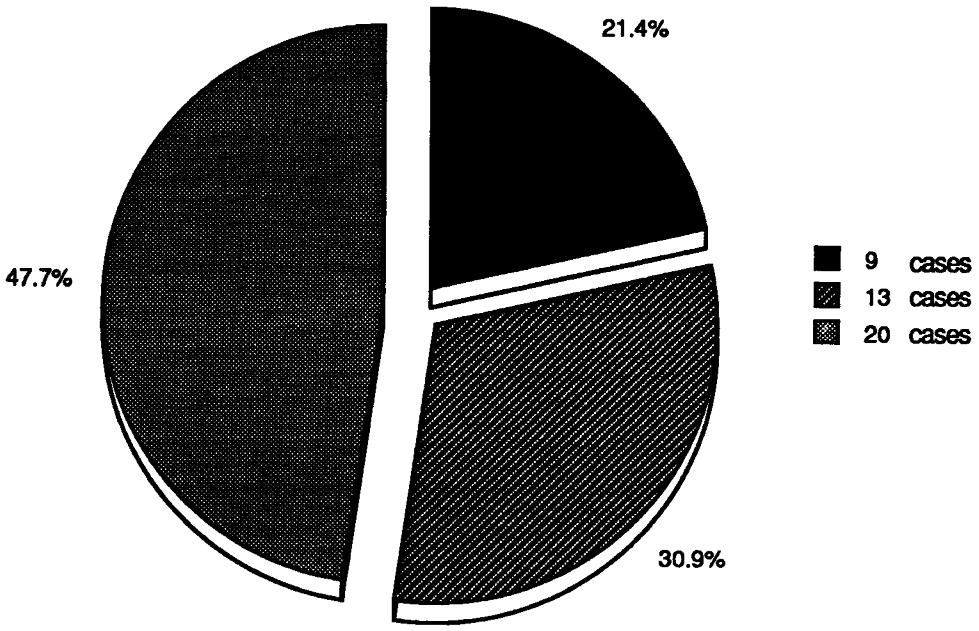

FIGURE 2 Patient distribution for three procedures performed by laparotomy: ■, unilateral salpingo-oophorectomy, 20 patients (47.7%); ■, bilateral cystectomy or unilateral cystectomy + wedge resection, 9 patients (21.4%), ■, unilateral cystectomy, 13 patients (30.9%).

TABLE II Operating Time, Hospital Stay, and Blood Loss with Laparoscopy Compared with Laparotomy

| | Laparoscopy (mean ± SD) | $n$ | Laparotomy (mean ± SD) | $n$ | $\chi^2$ test |
|---|---|---|---|---|---|
| Operating time (min) | | | | | |
| Unilateral cystectomy | 95.7 ± 19.9 | 7 | 72.5 ± 24.5 | 20 | $p > 0.05$ |
| Unilateral SO | 114.6 ± 40.3 | 16 | 67.7 ± 16.7 | 13 | $p > 0.05$ |
| Bilateral cystectomy or SO + wedge resection | 157.6 ± 43.7 | 7 | 86.1 ± 15.2 | 9 | $p > 0.05$ |
| Total | 120.3 ± 43.4 | 30 | 73.9 ± 21.6 | 42 | $p < 0.01$ |
| Hospital stay (days) | 5.7 ± 1.9 | 30 | 12.0 ± 2.9 | 42 | $p < 0.01$ |
| Blood loss (ml) | 18.2 ± 1.7 | 30 | 105.9 ± 84.3 | 42 | $p < 0.01$ |
| < 50 ml | | | | | 9 (21.4%) |
| 50 – 100 ml | | | | | 19 (45.2%) |
| 101 – 200 ml | | | | | 8 (19.1%) |
| 201 – 300 ml | | | | | 5 (11.9%) |
| > 300 ml | | | | | 1 ( 2.4%) |

SO, salpingo-oophorectomy.

($p < 0.01$). The final operating time for laparoscopy diminished to the time for laparotomy. This tendency was not observed for laparotomy (Fig. 4).

The hospital stay in the laparoscopy group was only 5.7 ± 1.9 days, but in the laparotomy group was 12.0 ± 2.9 days. The blood loss during laparoscopic surgery was small (18.2 ± 1.7 ml) compared with 105.9 ± 84.3 ml for laparotomy. Both the hospital stay and blood loss in the two groups (Table II) showed significant differences ($p < 0.01$).

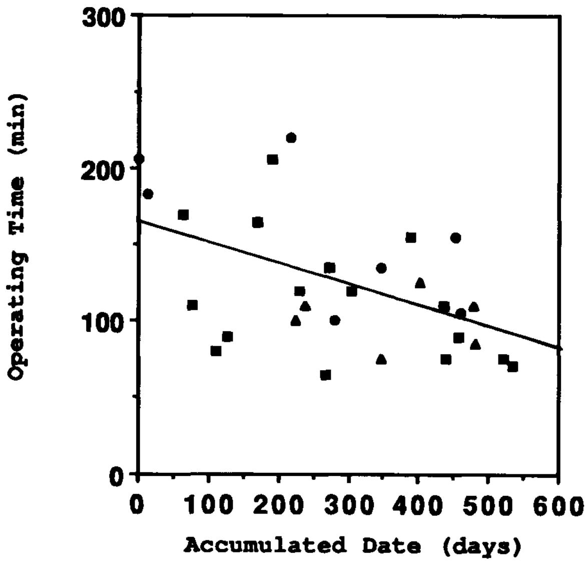

FIGURE 3 Correlation between operation time and accumulated date for three procedures performed by laparoscopic surgery:, ■, unilateral cystectomy, 16 patients; Δ, unilateral salpingo-oophorectomy, 7 patients, bilateral cystectomy, 7 patients; ●, bilateral cystectomy, 7 patients. Linear regression model for 30 patients combined: $p < 0.01$; $y = 164.00 - 0.13711x$; $r^2 = 0.249$.

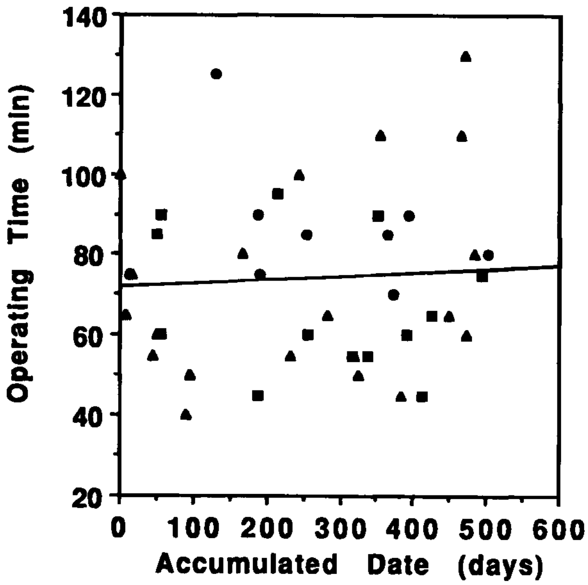

FIGURE 4 Correlation between operating time and accumulated date for three procedures performed by laparotomy: ■, unilateral cystectomy, 13 patients; Δ, unilateral salpingo-oophorectomy, 20 patients; ●, bilateral salpingo-oophorectomy or unilateral cystectomy + wedge resection, 9 patients. Linear regression model for 42 patients combined: $p > 0.05$; $y = 71.407 + 9.7062e - 3x$; $r^2 = 0.005$.

No intraoperative complications occurred. No short-term or long-term postoperative complications such as high fever or infection were found in either group. Spillage of cyst contents occurred in only one patient during the intraperitoneal laparoscopic procedure, and copious lavage was carried out immediately. There were no cases of secondary chemical peritonitis or conversion to laparotomy.

### DISCUSSION

Laparoscopic surgery has developed rapidly and widely, both in its application and technical instruments, but arguments against its use in the management of ovarian lesions still exist [11]. Although most reports have mainly focused on laparoscopic techniques, the potential advantages and feasibility of this procedure have been recognized. At present the major remaining controversies are the potential risk of malignancy after laparoscopic surgery [6], spillage of the cyst contents, and the potentially prolonged operating time [12]. In this study the mean operating times for the three kinds of laparoscopic procedures were longer than those for the same procedures performed by laparotomy; the mean time for laparoscopic surgery overall was also significantly longer than that for laparotomy. There are two possible reasons for this. First, it is acknowledged that when surgeons are learning new techniques, the time taken for the procedure will be somewhat longer; that is, there is a learning curve [13]. During this time the operator's skill improves, and eventually the operating time will become shorter. Our learning curve suggests that the operating time for each procedure in laparoscopic surgery fell remarkably with the progression of time, and almost reached that taken for laparotomy, but the coefficients of correlation for the three procedures showed no significant differences due to the small number of cases in each subgroup. When combining them, the curve not only showed a remarkable declining tendency but also a significant correlation coefficient. At the end of this period, the operating times taken for laparoscopic surgery were almost the same as those for laparotomy. We believe that operating times will become shorter in the near future with the continuous improvement in laparoscopic skill of our gynecologists and nursing staff. Another reason is that we work in a university teaching hospital where many younger surgeons perform only the first step of laparoscopy under the direction of the operator. The same situation also exists for nursing staff. We could not establish a special group to perform laparoscopic surgery, as was reported by others [14,15]. Laparoscopic surgery requires not only special surgical techniques but also familiarity with various complicated laparoscopy instruments. Furthermore, most surgeons completed their residencies before the widespread use of this procedure. They therefore need extensive retraining and study to take up this new approach, no matter how senior and proficient at open surgical techniques they are.

In most reports, the operating time for laparoscopic surgery in the management of ovarian cysts ranged from a minimum of 25 to 65 min to a maximum of 55 to 195 min with a mean of 40 to 95.5 min [9,13,14,16–21], which was not longer or was even shorter than that for laparotomy. Prolonged operating time was reported rarely and was due to the improper selection of patients. We agree that this new approach should be reserved for special surgical teams trained in gynecologic oncology and major laparoscopic surgery to ensure efficiency and safety [7]. In addition, gynecologists and nursing staff should undergo retraining in laparoscopic surgery to master this new technique. Only in this way can laparoscopic surgery gain acceptance.

This study also showed a significantly shorter hospital stay and less blood loss in the laparoscopy group compared with the laparotomy group. These are the major advantages of laparoscopic surgery, suggesting that the optimal surgical goal can be achieved with a minimum of invasive procedures and injury to patients. The recuperation time was shorter, regardless of the extent of the procedure owing to the small abdominal incisions. Some authors reported that their patients could be discharged 24 to 48 hr after surgery [2,14,17]. However, we do not advocate early discharge, since we consider that the procedures are the same as those performed via laparotomy, and further observation is indicated for safety. More clinical trials may be required to confirm this. Another important advantage of laparoscopy is the decreased overall cost, which was reported by others [2,22]. However, we did not investigate this issue in the present study.

All reports about the spillage of tumor contents during surgery suggest that it should not adversely affect the prognosis of patients [23]. A well-designed prospective study is urgently required to confirm this. Until then, we should endeavor to prevent spillage of tumor contents, which is the most important part of the technique in laparoscopic surgery for ovarian dermoid cyst. Many methods of retrieval to prevent spillage of tumor during laparoscopic surgery have been reported, such as using the Endo Pouch [16,21] or Endocatch [18]. The zipper storage bag [15] is convenient if the tumor is larger than 10 cm and has the lowest cost. As the zipper bag is a fixed size, sometimes it may be too large to use for smaller tumors. We reported our experience using an isolating bag, which could be cut according to the tumor size. The bag is cut in a triangular shape with a wide opening and a small amount of saline is put in it to make it easy to open the bag and move the tumor into it in the peritoneal cavity. A large tumor could be easily removed by aspirating the tumor contents without any contamination of the operating field. The risk of tumor spillage could thus be minimized. The laparoscopic manipulation requires considerable expertise. From our experiences, we consider that a well-designed bag in several sizes with low cost is desirable.

The most controversial issue at present is the potential risk of subsequent malignant tumor after laparoscopic surgery. Based on the survey of the AAGL in 13,739 cases of laparoscopic ovarian cyst surgery, 53 (about 0.04%) were found to have evidence of malignancy [1]. The incidences reported by others were 0.39% [24], 0.9% [25], and 1.4% [26], which were much lower than we anticipated. We did not experience such a case, which may be due to the small number of patients included in this study. However, we consider that the strict selection criteria we used did play an important role. In patients older than 60 or younger than 20 years, we did not consider the use of laparoscopy since the risk of malignant change is markedly higher in these two age groups [27]. In patients with markedly elevated levels of tumor markers, laparoscopic surgery was also not performed, even though elevated levels of CA125 and CA19-9 are generally found in cases of ovarian dermoid cyst. We also examined high-quality frozen sections if there were any signs suspicious of malignancy. The preoperative diagnosis is not 100% accurate, both for laparoscopic surgery and laparotomy cases. Ovarian cancer in a normal-sized ovary is not uncommon, and the possibility of neglecting this kind of malignancy exists even at laparotomy. If a malignancy is found after laparoscopic surgery, laparotomy should be performed as soon as possible to confirm the stage of the disease to determine further treatment. We consider that both laparoscopic surgery and laparotomy provide good access for the treatment of ovarian tumors, and each has its own benefits and pitfalls. Although laparoscopic surgery has many advantages over laparotomy in the treatment of some types of ovarian lesions, it cannot replace laparotomy in all cases. The method that achieves the best surgical results with the least invasive approach and complications is the best procedure for the patient. The principles of laparoscopic surgery, which have been described many times by the pioneers of laparoscopy [3] should always be followed to apply this new approach more effectively and safely.

We conclude that this study again demonstrated the efficiency and safety of laparoscopic surgery in the management of ovarian dermoid cyst. The advantages of operative laparoscopy such as a short recuperation time and hospital stay, less blood loss, lack of complications, and less invasive procedures were shown in this study. Strict criteria for patient selection should always be followed, and retraining schedules for gynecologists are essential.

### *References*

[1] Hulka, J. F., Parker, W. H., Surrey, M. W., *et al.* (1992). Management of ovarian masses: AAGL 1990 survey, *J Reprod Med*, **37**, 599–602.

[2] Garry, R. (1992). Laparoscopic alternatives to laparotomy: a new approach to gynecological surgery, *Br J Obstet Gynecol*, **99**, 629–632.

[3] Steege, J. F. (1994). Laparoscopic approach to the adnexal mass, *Clin Obstet Gynecol*, **37**, 392–405.

[4] Koonings, P. P., Campbell, K., Mishell, D. R., *et al.* (1989). Relative frequency of primary ovarian neoplasms: a 10-year review, *Obstet Gynaecol,* **74**, 921–926.
[5] Peterson, W. F., Prevost, E. C., Edmiends, T. T., *et al.* (1955). Benign cystic teratoma of the ovary: a clinico-statistical study of 100 cases with review of the literature, *Am J Obstet Gynecol,* **70**, 368–382.
[6] Maiman, M., Seltzer, V., Boyce J. (1991). Laparoscopic excision of ovarian neoplasms subsequently found to be malignant, *Obstet Gynecol,* **77**, 563–565.
[7] Maiman, M. (1995). Laparoscopic removal of the adnexal mass: the case for caution, *Clin Obstet Gynecol,* **38**, 370–379.
[8] Hasson, H. M. (1974). Open laparoscopy: a report of 150 cases, *J Reprod Med,* **12**, 234–238.
[9] Ikuma, K., Shiotani, T., Shibahara, H., *et al.* (1992). Laparoscopy guided extraperitoneal resection of ovarian cysts: surgical procedure, indications and limitations, *Acta Obstet Gynaecol Jpn,* **44**, 1529–1536.
[10] Manyonda, I. T., Baggish, M. S., Bower, S., *et al.* (1993). Combined laparoscopic and micro-laparotomy removal of benign cysticteratoma, *Br J Obstet Gynecol,* **100**, 284–286.
[11] Pitkin, R. M. (1992). Operative laparoscopy: surgical advance or technical gimmick, *Obstet Gynaecol,* **79**, 441–442.
[12] Selizer, V. (1994). Laparoscopic surgery for ovarian lesions: potential pitfalls, *Clin Obstet Gynecol,* **36**, 402–412.
[13] Yuen, P. M., Rogers, M. S. (1994). Laparoscopic management of ovarian masses: the initial experience and learning curve, *Aust NZ J Obstet Gynaecol,* **34**, 191–194.
[14] Nezhat, C., Wendy, K., Winter, R.N., *et al.* (1989). Laparoscopic removal of dermoid cysts, *Obstet Gynaecol,* **73**, 278–280.
[15] Yuen, P. M., Rogers, M. S. (1994). Laparoscopic removal of ovarian cysts using zipper storage bag, *Acta Obstet Gynecol Scand,* **73**, 829–831.
[16] Hasegawa, I., Tanaka, K., Utsumi, T., *et al.* (1994). Laparoscopic removal of ovarian dermoid cysts using specimen retrieval bag, *Acta Obstet Gynaecol Jpn,* **46**, 145–147.
[17] Herendael, B. V., Beretta, P., Slangen, T., *et al.* (1995). Management of adnexal masses by operative laparoscopy, *J Am Assoc Gynecol Laparosc,* **2**, 273–277.
[18] Ishikawa, H., Ito, M., Goto, M., *et al.* (1994). Laparoscopic extirpation of ovarian tumor: a trial to prevent spillage of tumor contents, *Acta Obstet Gynaecol Jpn,* **46**, 607–610.
[19] Perry, P. C., Upchurch, J. C. (1990). Pelviscopic adnexectomy, *Am J Obstet Gynecol,* **162**, 79–81.
[20] Reich, H., McGlym, F., Sekel, L., *et al.* (1992). Laparoscopic management of ovarian dermoid cysts, *J Reprod Med,* **37**, 640–644.
[21] Yuen, P. M., Rogers, M. S. (1993). Laparoscopic removal of dermoid cysts using Endo-pouch, *Aust NZ J Obstet Gynaecol,* **33**, 397–399.
[22] Levine, R. L. (1985). Economic impact of pelviscopic surgery, *J Reprod Med,* **30**, 655–659.
[23] Parker, W. H. (1995). The case for laparoscopic management of the adnexal mass, *Clin Obstet Gynecol,* **38**, 362–369.
[24] Nezhat, F., Nezhat, C., Welander, C. E., *et al.* (1992). Four ovarian cancers diagnosed during laparoscopic management of 1011 women with adnexal masses, *Am J Obstet Gynecol,* **167**, 790–796.
[25] Canis, M., Mage, G., Pauly, J., *et al.* (1994). Laparoscopic diagnosis of adnexal cystic masses: a 12-year experience with long term follow-up, *Obstet Gynecol,* **83**, 707–712.
[26] Mecke, H., Lehmann-Willenbrock, E., Ibrahim, M., *et al.* (1992). Pelviscopic treatment of ovarian cysts in premenopausal women, *Gynecol Obstet Invest,* **34**, 36–42.
[27] Mann, W. J., Reich, H. (1992). Laparoscopic adnexectomy in postmenopausal women, *J Reprod Med,* **137**, 254–256.

